# Application of Innovative Methods of Predictive Control in Projects Involving Intelligent Steel Processing Production Systems

**DOI:** 10.3390/ma14071641

**Published:** 2021-03-27

**Authors:** Jozef Svetlík, Peter Malega, Vladimír Rudy, Ján Rusnák, Juraj Kováč

**Affiliations:** 1 Department of Manufacturing Machinery and Robotics, Faculty of Mechanical Engineering, The Technical University of Košice, Letná 9, 040 01 Košice, Slovakia; 2 Institute of Management, Industrial and Digital Engineering, Faculty of Mechanical Engineering, The Technical University of Košice, Letná 9, 040 01 Košice, Slovakia; peter.malega@tuke.sk (P.M.); vladimir.rudy@tuke.sk (V.R.); juraj.kovac@tuke.sk (J.K.); 3 U. S. Steel Košice, s.r.o, Vstupný areál U. S. Steel, 044 54 Košice, Slovakia; janrusnak2@sk.uss.com

**Keywords:** specific measurable assignable realistic time-related (SMART) quality assurance, rolling mill, steel strips, rolling mill defect verification, pickling line, continuous high-capacity strip pickling, strip tear trigger

## Abstract

This paper describes the enhancement of the existing predictive system of quality management in the processes of metallurgic manufacturing. Specifically, it addresses steel-strip manufacturing. The main quality management innovation is the transition from the current methodological process of a single-step defect evaluation to a two-step evaluation. A two-step defect check of the strip’s surface involves checking for defects during the hot-rolling process first, and double-checking it during the process of pickling. These defects are detected in a well-established process of camera imaging in the production process. The recorded image is then processed mathematically to find the degree of defect correlation in those processes. The two-step evaluation enables a more detailed focus on a particular defect and its position on the strip. Decisions concerning further processing are based on defect evaluation, for instance, whether a rework is necessary to maximize the product utilization and minimize the eventual negative impact of the defect on production equipment. A crucial aspect is also the reduced probability of failures in the manufacturing process.

## 1. Introduction

The implementation of the elemental industry 4.0 tools in the metallurgical manufacturing process is taken for granted. These tools apply the specific measurable assignable realistic time-related (SMART) quality assurance principles.

The team led by Samsudin [1] proposed a method that can be used with the local binary pattern to sort the images into six defect classes according to the most common defects found on the rolled steel surfaces.

Research undertaken by Woo, et al. [2] looked into the shape defects originating in the flexible-roll-forming process of double-layered slabs made of steel/aluminum. Three slabs of different shapes—namely the trapezoidal, concave and convex ones—were examined for such defects as web-warping, wrinkling, and delamination at the interface. The results showed that web-warping occurred in both single- and double-layered slabs, while only double-layered slabs were affected by wrinkling caused by the compressive longitudinal stress acting on the flange of the convex slab.

Authors Zhang et al. [3] proposed a new, fuzzy defect detection method aimed at sets. Unlike the traditional methods of defect detection, this method uses a membership function to gauge if any particular grey level may be a suspected defect. The method can show defects with a great degree of precision when combined with pixel connectivity.

Research that was conducted by Liu, L. M. et al. [4] resulted in a novel classification model that improved the least-squares support vector machine. It has found its application in detecting defects on the surface of steel plates. Experiments have proven this model’s advantage lying in its high speed and a good degree of correct classification, particularly for corrupted defect datasets.

In their research, Lu et al. [5] examined microcomputed tomography as a validation method of correlating features observed in optical images. They found that features detected on the spot could be matched with defects detected by microcomputed tomography, thus revealing the possibility of using optical images as a means of defect detection, in their case, in the parts that were selected for melting by laser.

Research conducted by the authors Mentouri et al. [6] employs the method of Binarized Statistical Image Features, which was used in biometrics until now. A higher average rate of recognition has been achieved in this study, namely of six types of defects occurring in the hot-rolling process. The lower standard deviation pointed to its robustness and an acceptable sample processing time. Filters used in this study provide a quite discerning description of images. Data were reduced to include the relevant ones, and the applied classifier proved efficient in recognizing hot-rolled products featuring a defect at an acceptable rate.

The team led by Lu [7] proposed a method of defect segmentation for discrete Fourier spectral residuals and Gabor filtering image fusion to address the issue of segmenting surface defects on galvanized steel against a complex textural background. The team has demonstrated that the proposed improved spectral residual-Gabor method overcomes the traditional Gabor method in its segmentation accuracy and detection of surface defects of galvanized sheets. The experiment results show that this enhanced method is highly accurate in segmenting the defects and may, to a certain degree, be used as a benchmark method for examining the flaws of zinc-coated sheets.

Four authors, Pawar et al. [8] used techniques of surface description, namely the Raman spectroscopy and Fourier transform infrared spectroscopy, plus the scanning electron microscopy–elemental dispersive spectroscopy (SEM-EDS), for their analysis of defective versus defect-free regions of steel sheets. They have found that the cold-rolled steel sheets were contaminated by grease at the electrolytic cleaning line (ECL) entry, which resulted in a defect demonstrated as white patches. To prevent such contamination in the future, periodic cleaning and preventive maintenance of plant machinery were recommended.

In their scientific paper, Zhang et al. [9] proposed a method of feature selection using filtering in combination with hidden Bayesian classifiers. This resulted in improved defect detection and reduced complexity of calculation. The method is capable of selecting an optimal hybrid model capable of accurate steel-strip surface defect classification. The importance of this paper lies in the development of a hybrid model that combines algorithms for feature selection and multi-class classification of steel-strip surfaces.

A trio of authors, Kikuchi et al. [10] has conducted research into the possibilities of how microdefects, such as cracks, can be detected in steel. Their method of choice was the magnetic flux leakage method. With the help of a giant magnetic impedance sensor, they detected a 100-μm defect through the application of a weaker field. Detection sensitivity was limited by the sensor size and its dynamic range. In addition, a gradiometer consisting of giant magnetic resistance elements was able to detect a defect as small as 30 μm in diameter.

Research by Zhou et al. [11] presents a model of convolutional neural networks deployed to the task of steel sheet surface defects classification. Unlike other methods, this one is capable of achieving simultaneous feature extraction and classifier design. The experiment on a small dataset, using a small model, has demonstrated moderate accuracy in sheet steel surface defect classification; the average classification accuracy can be as high as 99%.

Li et al. [12] compiled a dataset of six cold-rolled steel-strip surface defect types, which they augmented to reduce over-fitting. This research team improved the You Only Look Once (YOLO) network and made it all convolutional. The improved network, consisting of 27 convolution layers, is a comprehensive solution to detecting steel-strip surface defects. They applied their network to the six defect types and achieved 97.55% mAP performance and a 95.86% recall rate.

In the research presented by Hu et al. [13], a classification model based on a support vector machine constructed for the purpose of classifying the defects found in the steel-strip surface images has employed the geometric, the grayscale and the shape features extracted from the combination of the target defect image and its matching binary image. Gauss radial basis was used as the kernel function, the model parameters were selected by cross-validation and a *one-versus-one* approach was employed to multiclass classification. A higher average classification accuracy has been achieved by the support vector machine model than by the model based on the classic back-propagation neural network, with an optimal number of hidden layer neurons assigned to it.

Authors Cięszczyk et al. [14] proposed a method of steel defects detection utilizing a fiber Bragg grating sensor. They have also proved the usefulness of their method experimentally. The defects examined were grooves periodically occurring along the tested steel-strip length. The fiber spectral reflectance properties were measured directly, solving the corresponding inverse problem of establishing indirect defect shape.

In the paper written by the team led by Zhang [15], the goal was a reduction in the number of sheet steel defects through a change in the operating conditions, specifically in the variables identified as impactful. To predict the number of defects in the casting-rolling process online, a defect prediction model was used as a virtual sensor. Informed by the online prediction of the number of defects, operators can take suitable measures (like changing the operating conditions or stopping the operation if the requirements for the output are not met).

Authors Kang et al. [16] proposed the steel-strip surface defects be detected by the feed-forward neural network. They proceeded from the premise that any defect reconfigures the arrangement of the surrounding pixels, so they were able to extract the feature vectors in this way. The feature vector size was reduced in the principal component analysis using singular value decomposition. After this, steel-strip defects were detected by the feed-forward neural network.

A trio of authors, Wang et al. [17], developed an online method of detecting 3D defects based on the photometric stereo. This enabled them to accurately identify and locate 3D defects in sheet steel. For steel passing through at high speed, a photometric stereo laser scanning system was designed for online detection. By avoiding the “pseudo-defect” disturbances, the detection accuracy has significantly increased.

The team led by Sun, Q. [18] developed a new method for detecting and locating the steel-strip surface defects on the basis of Singular Value Decomposition. The beauty of this method lies in easy and accurate detection and location of common defects. The method reliably establishes not only the steel-strip surface defects but also their approximate location on the strip. This defect detection method is based on direct image processing, without its pre-processing by segmentation or modelling.

Authors Liu et al. [19] proposed a defect classification method based on deep convolutional neural networks (CNNs). They started with the GoogLeNet base model and added the mechanism of identity mapping for an additional improvement. They also compiled a six-type dataset of cold-rolled steel-strip surface defects and augmented it to reduce over-fitting. They reached an accuracy of 98.57%.

A paper from a trio authors, Suvdaa et al. [20], described a new framework for steel surface defect detection and classification. This new approach adopted scale-invariant feature transform for defect point detection. Through this algorithm, authors can easily increase the number of training samples for support vector machines. Experimental results demonstrated the efficiency of this approach in detecting and classifying steel surface defects.

Research undertaken by Mancini et al. [21] described the use of standard and advanced modelling techniques to resolve industrial problems experienced in hot-rolling. To examine defect formation, the material has been first assigned mechanical characteristics. In addition, metallurgical models with the encompassed theory of material damage have been implemented and coupled with FE calculation results for the sake of predicting the microstructural evolution of the ferrite grain structure in the hot deformation process. Results point to defect causation induced by the processing conditions that trigger anomalous heat build-up, which, in turn, induces an uncontrolled grain growth at the edges.

Steel plate defect identification has been tackled by Tian et al. [22] by means of their improved Extreme Learning Machine algorithm named Genetic Extreme Learning Machine. This algorithm employs certain additional mutation rules that are capable of offsetting the randomization uncertainties of the original algorithm. Experiment results involving nine typical defect samples showed that the Genetic Extreme Learning Machine algorithm effectively improved the identification accuracy of the Extreme Learning Machine algorithm.

A research team led by Xie et al. [23] came up with a new approach to establishing the size of subsurface defects of metallic sheets by square pulse thermography. The tool of their choice was the oriented gradient of histograms. The ability of the proposed approach to establishing the size of both the distinct and the blurry defects with high accuracy across repeated detections by the square pulse thermography of metallic plates was confirmed experimentally.

In research done by Chu et al. [24], the authors proposed the sheet steel surface defects be detected through multi-type statistical features and an enhanced twin support vector machine. Dummy boundary samples and representative samples were used to build the classification model. Both the dummy boundary and the representative samples have experimentally proven to improve the accuracy and efficiency of the multi-class classifier. The proposed multi-type statistical features, together with the enhanced twin support vector machine, have demonstrably improved the defect recognition performance in comparative experiments.

Research conducted by Liang et al. [25], addressing the sheet steel surface defect classification, proposed an SP-DenseNet framework of classification based on the combination of self-paced learning and DenseNet. To detect the surface defects occurring on sheet steel, a special self-paced process has been designed. To successfully address the quality problem, the framework draws on the concept of self-paced learning that makes the detection results more reliable.

Aspects of a fast-moving object’s macrostructure digitization are analyzed in paper [26]. In addition to particular imaging equipment features, the authors also explore the options for recording the geometric surface profile. An important parameter subject to examination was to record the surface deviations with sufficient precision and the ability to process the obtained digitized data in the group for optical metrology (GOM) inspect software for surface analysis.

The substrate entering the manufacturing process, i.e., the rolls designated for the rolling process according to the technological requirements, is currently not accompanied by the data from the preceding production processes in digital form, enabling its evaluation by the rolling process control system. Records of the preceding process come as the accompanying documentation in printed form is processed by the team manning the production line. This documentation contains records of eventual defects found in the production of the given roll, evaluated in the Hold System. The role of this system is to intercept those rolls in the production process that deviate from the required quality parameters. When these deviations are removed, they are released from the Hold System for further processing.

A high-precision production system generates massive data flow [27]. It is necessary to organize the sorting and mutual communication of this data. In this case, the data is divided into three essential levels based on its generation, direction and the information it contains:

Level A—represents data obtained directly from the process, and processed in the processing system, via sensors, readers and related instruments.

Level B—ensures that the technical requirements are met, the process requirements are transferred to the process. It reads, monitors and controls physical processes.

Level C—provides output for checking the processes, enables their analysis in the production flow. Its purpose is to function as the production qualitative parameters tool.

It is usually possible to use a variety of virtual models at the general level of the production system control. Becoming an ever more important aspect of production is the issue of project management optimization. To illustrate the point, let us present at least one example [28].

The presented two-step defect verification system in metallurgic manufacturing is innovative compared to conventional methods of quality monitoring. The surface quality is evaluated in the direction of defect confrontation detected on the strip surface in two subsequent manufacturing processes. First, in the hot-rolling process and subsequently at the output from the pickling process. Defects detected and classified on the hot strip surface are eliminated in the pickling process in some cases, which is why they do not pose a risk in production flow at a later stage. For example, in the rolling process, when critical defects remain on the strip surface, it is necessary to reconsider the further processing of such strips.

The conventional surface defect evaluation process did not examine the correlation between the surface defects of two consecutive manufacturing processes. Likewise, the option of eliminating negative influences on the final product and eventual failures caused by those defects have not been examined either. As an example, strip rupture in the rolling process and many other critical events in the final production aggregates may be cited.

### 1.1. Production Systems Communication Interfaces

An ISA-95 standard [29] has been developed for global manufacturers, which is applicable to the systemic communication of the automated interfaces in different industries and industrial processes, suitable for many continuous and repetitive industries and process types. This is usually the case for high-volume flow production.

This standard enables the transfer, implementation, and production process management in accordance with the client’s order. Purchase contract information, individual technological parameters, and support processes are implemented at the respective levels of the manufacturing process:

- Level 0—defines physical processes
- Level 1—smart devices which read and control physical processes
- Level 2—control systems software manages the process in real-time, using a mathematical model
- Level 3—production support control data, its function is to support the production control
- Level 4—production process logistic control of the data flow

### 1.2. Digital Imperfection Registry

The digital imperfection registry records the deviations from the prescribed parameters in digital form [30]. They are created at the moment of casting the slab in the continuous slab casting works and accompany the slab throughout the production process, all the way to its shipment. The slab digital imperfection registry data contain records of each production operation, and they are relayed to the production equipment’s control system. Based on the incoming data, the production equipment ensures the process settings meet the required parameters, taking into account the eventual deviations from the required parameters of the incoming substrate. The goal is to eliminate deviations during the course of the process and to achieve the parameters expected. The incoming rolls’ imperfection data enters the entry looper together with the batch of incoming rolls. The data is continuously evaluated, and a quality control frequency calculation is made from defect indications on the rolls designated for rolling. The process of quality control frequency calculation (1) takes into account the following correlation:

- The number of rolls that enter the process and are put on hold;
- Defect severity;
- Empirical coefficient (a coefficient know from practice; it evaluates the consequences of a defect evaluated in the past);
- The unit of calculation is the number of quality controls per roll designated for rolling.

(1) $$FQC\;=\;\frac{10}{\left(M\;+\;S\right)\;\times \;EC}$$

*FQC*—quality control frequency, i.e., the number of rolls that pass down the line until the next roll inspection [number of rolls/control]

*M*—Multiplicity—the number of defects occurring per 10 rolls entering the process, which means the number of defects found in the batch of 10 consecutive rolls entering the rolling process. [number of defective rolls/10pcs]

*S*—Severity—the severity of the defect on the strip, ranging from 1, which is the least severe defect, all the way up to 10. [dimensionless number]

*EC*—Empirical Coefficient—practical coefficient represented by a value obtained from past experience. If the defect caused a small number of ruptures in the rolling process in the past, 0.1 is used. The value of 0.2 is selected for moderately common ruptures, or 0.3 is used as the highest value representing frequent adverse consequences in the attempt at reworking the roll (dimensionless number).

The example describes the calculation of the FQC for ten rolls with defects and defects that are critically severe. However, this is done under the assumption that these defects were successfully eliminated in the rolling process on the respective line. We enter these conditions into Equation (2).

(2) $$FQC\;=\;\frac{10}{\left(10\;+\;10\right)\;\times \;0.1}\;=\;5\;[\mathrm{of}\;\mathrm{rolls}/\mathrm{inspection}]$$

where:

multiplicity = 10 [number of defective rolls/10pcs]

severity = 10 [dimensionless number]

empirical coefficient = 0.1 [dimensionless number]

It follows from the result (2) that under the stated conditions, it is appropriate to carry out a detailed inspection of every fifth roll.

If the result of the roll’s quality control is negative, the strip continues to the rolling process without a corrective intervention. The next quality control calculation takes place after the designated number of rolls has undergone the rolling process.

Should the quality control yield a positive result, the rolling mill operators embark on corrective measures to remove the source of poor quality (e.g., roll replacement). When the source of the poor quality has been replaced, quality control is conducted to check the effectiveness of the corrective procedures. At the same time, the system of calculation determines the subsequent number of the rolls undergoing the rolling process before the next inspection.

The defect detected by the quality control is logged into the digital roller imperfections registry. Two processes take place simultaneously. The first is entering the defect in the Hold System, tracking it all the way back to the last quality control. The second process, the SMART control system, charts the most suitable course of the detected defect’s further processing.

### 1.3. Predictive Quality Control in the Strip Pickling Process

The hot strip parameters’ divergence from the reference parameters has a crucial impact on the pickling process. The hot strip’s exposed parameters are the following:

- strip geometry
- surface defects
- mechanical properties
- deviations in dimensions

**Figure 1:**
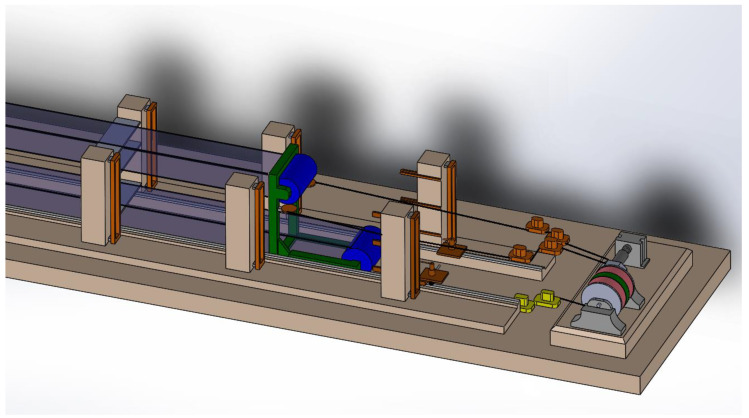
Continuous pickling line looper. The annual capacity is 1,200,000 tons; the looper capacity is 1150 m; the maximum strip width is 1600 mm; and the maximum thickness is 5 mm.

**Figure 2:**
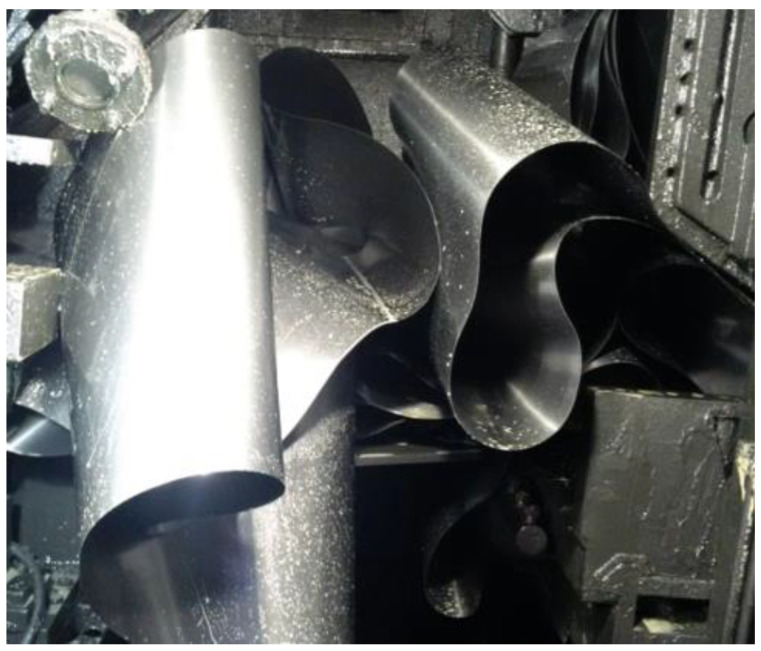
Consequences of strip rupture in the cold-rolling process.

The hot strip geometry has a critical effect on guiding the strip in the pickling process. The strip guiding in the pickling line has a crucial impact on the pickling line’s performance, Figure 1. Should the hot strip geometry be non-compliant, there is a probability of the strip’s yaw during the pickling process, and undesirable events, such as the strip’s rim damage, may occur. The strip rim damage is inadmissible, as it may result in the strip’s rupture in the pickling process, and, in addition, a damaged strip rim poses a high risk in the subsequent production process, mainly during that of the cold rolling. The pickling lines feature a device that keeps the strip aligned with the line axis. These devices normally ensure that the strip maintains the desired position on the line. If the hot strip’s parameters diverge significantly from the reference parameters, a situation involving the strip yawing from the safety zone may occur. The consequences of the strip’s yaw are highly undesirable, as shown in Figure 2.

High-performance continuous pickling lines feature loopers, Figure 1, enabling continuous high-capacity strip pickling. Deviation from desirable geometry poses a risk. The highest risk is exactly in those loopers. The loopers follow the motion of the looping wagon, moving in the looper space, which results in the strip hanging over between the steering roller and the looping wagon. The strip’s position on the looping wagon is directly proportionally correlated with the strip’s distance from the steering roller and from the separators’ geometry. The default setting of the separators’ geometry matches the reference parameters of the hot strip geometry. If the hot strip geometry diverges, the strip yaws in the looper.

This adverse phenomenon under scrutiny may be eliminated through reduced looper load. If a strip with unsuitable planar parameters is processed and there is a heightened risk of the strip yaw in the pickling line, a SMART production requires data to be imported with the parameters’ level of deviation from the reference values of the strip geometry and the strip’s down web-position.

A PDI data telegram in Appendix A is uploaded from Level B, and it includes the standard data in addition to the descriptive data of SMART production. It is necessary that it also contains the data on the strip’s planar divergence, as discussed above. Based on the Level B data, the Level 1 control manages the production process for performance optimization and the minimization of the risk of rupture in the production process. This predictive system regulates the looping wagon’s position, basically correlating with the planar deviation level. The planar deviation culminates in time synchronization of the wagon’s position and the critical strip position. The aim is to minimize the looper’s load. This significantly reduces the probability of a critical event due to a looping wagon failure.

### 1.4. Surface Defects

The pickling process surface defects may be divided as follows:

- Those entering the pickling process
- Those emerging in the course of the pickling process

Defects entering the pickling process emerge in the processes preceding the pickling process. Crucial to the pickling process are the defects that emerge in the process of continuous slab casting and in the hot-rolling process. To satisfy the basic notion of predictive production management, data transfer between individual production processes in the production flow is crucial.

The continuous slab casting also gives rise to defects that can be eliminated, or their adverse effects can be significantly reduced in the subsequent production flow, subject to their detection, monitoring, and removal in the suitable process operating during production on the various machinery. Defects emerging in the process of continuous slab casting are the following:

- Non-metallic inclusions
- Slag nests
- Metal protrusions
- Cracks
- Rolled-in foreign material
- Transversal slab
- Scratches
- Slivers

Defects emergent in the process of slab casting tend to change their shape and position in the hot-rolling process, thus making it possible for the defects to submerge under the strip surface and give rise to a latent characteristic. Such latent characteristics pose a risk in the subsequent processes, e.g., a failure, and of course, it significantly affects the finished product quality.

**Figure 3:**
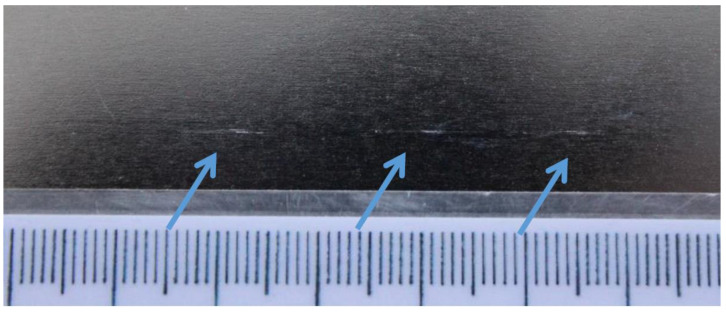
Scratches resulting from the steel-strip cold-rolling process.

The following defects emerge in the hot-rolling process:

- Scales
- Scale scars
- Imprints
- Scratches, Figure 3
- Pincher

Innovative project methods of metallurgic production lines predictively calculate and project data flows. They do so in conjunction with the flow of material and the transfer of particular divergencies in quality in order to eliminate the latter in the subsequent flow. It is exactly the pickling process that necessitates that surface defects, emergent in the hot-rolling process, and all their respective parameters, be precisely defined as follows:

- Defect type
- Position
- Recurrence
- Scale

Data were distributed based on the defect parameters’ exact definition in the PDI telegram, Figure 3, and the pickling parameters were set accordingly to eliminate the defects. The pickling process makes it possible to eliminate some, but not all, of the defects from the preceding processes.

**Figure A1:**
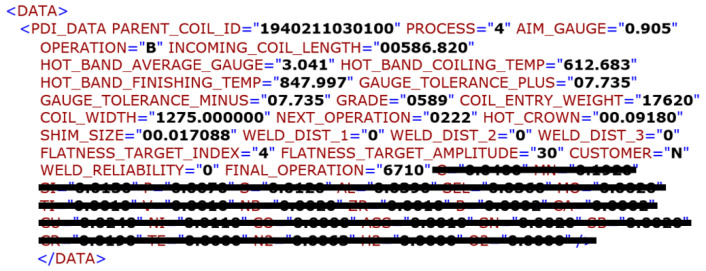
An example of a PDI data telegram in the HTML format from the cold-rolling mill. Four-seat tandem rolling mill. Initial strip thickness: 3.041 mm; exit thickness: 0.905 mm. Technological steel code 589, strip width: 1275 mm. Roll weight: 17,620 kg; strip coiling temperature at hot-rolling plant: 612 °C.

Rolled-in scales emergent in the hot-rolling process may be eliminated if the pickling process speed is reduced. The PDI telegram, Figure A1 in Appendix A, transferred to the pickling process, contains exact data on the scale’s occurrence position. It is required that the pickling process speed be reduced at the place of the scales’ occurrence. At the same time, the pull breaker parameters are exposed, causing disintegration of the scales layer, thus increasing the pickling process efficiency. The elimination result is checked at the output from the pickling line via a camera system.

## 2. Materials and Methods

**Figure 4:**
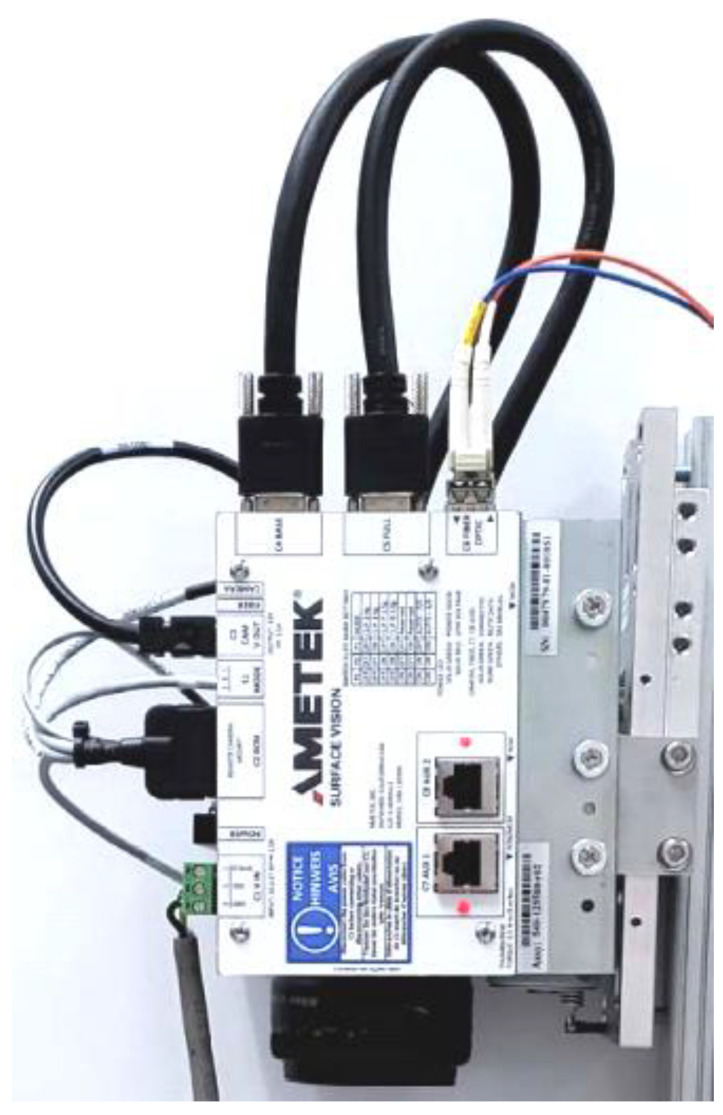
Surface inspection camera with Ametek Camera Link Interface module [31].

Defects in the steel-strip processed in the pickling line are detected primarily by camera systems, such as, for example, Figure 4. This figure shows the camera with a Camera Link Interface module (AMETEK Surface Vision, Hayward, WI, USA) installed at the pickling line [30].

The SmartView system uses line scan cameras to continuously inspect the moving web and detect defects in the material. These cameras feature a group of light sensors (picture elements or pixels) that build up an electrical charge proportional to the light that reaches them (called exposure). The pixels are then digitized and shifted out of the camera. This process is repeated thousands of times per second. The rate at which this is done is called the camera scan rate and is measured in line scans per second.

The camera works at 320 MHz speed and a 6 k resolution, which translates into a 53.3 kHz line rate. But there is a limitation on the SPU side, allowing the processing of only 320 megapixels (understood as MHz) per second, so the camera speed is reduced to the maximum of 160 MHz (26.6 kHz line rate) to cover two cameras.

**Figure 5:**
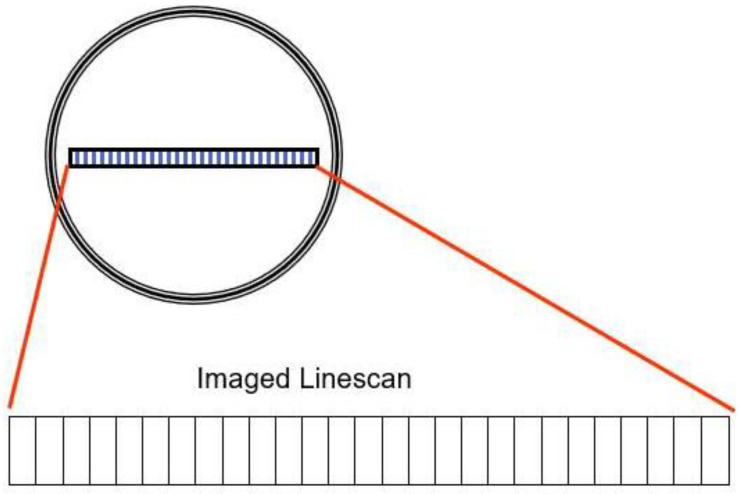
The array of SmartView camera, with a single row of pixels [30].

Some cameras, such as those used in modern digital cameras, have a group of pixels arranged in rows and columns, forming a rectangular shape (like the rectangular film of a standard photographic camera). Figure 5, however, shows SmartView cameras that have a single row of pixels and are, therefore, called line scan cameras because they see (scan) one line at a time.

The camera system supplier claims an 85% success rate in defect classification. Our real-life experience shows that the accuracy of the proposed system is somewhere immediately below the declared accuracy of this camera system.

Based on the requirement to reduce the number of ruptures in the rolling process, the SMART production tools were implemented with the goal of interconnecting the data obtained in individual processes and of utilizing their potential in the production process. Strip destruction is a frequent occurrence in the cold-rolling process, resulting in severe damage caused by such incidents. Accordingly, a methodology for analyzing the causes and the effects of ruptures was compiled. Based on the designed procedure, a ruptures database was created and analyzed for strip surface defects detected in the pickling process.

In terms of defect evaluation, the following parameters were assessed:

- Defect type
- Distance from the strip edge
- Position along the strip’s length
- In multiple occurrences, also distance from the closest defect
- Bisurfacity of the defect, should it reach all the way to the opposite side
- Steel grade
- Defect size
- Defect density

The secondary but not less important task was to analyze the rolling process for a defect’s potential to cause the strip rupture in the rolling process. The following input parameters entered this evaluation process:

- Reduction value
- Rolling speed
- Values of the rolling forces in the rolling process
- Reduction at individual seats of the rolling mill

## 3. Results

Stemming from the requirement to reduce the number of ruptures in the rolling process, SMART production tools were implemented with the aim of interconnecting the data obtained in the respective processes and of utilizing their potential in the production process. Strip destruction frequently occurs in the cold-rolling process, resulting in severe damage caused by such incidents. A method for analyzing the rupture causes and effects was designed accordingly. Based on this method, a ruptures database was created and analyzed in terms of strip surface defects detected in the pickling process.

### 3.1. Established Rupture Triggers

**Figure 6:**
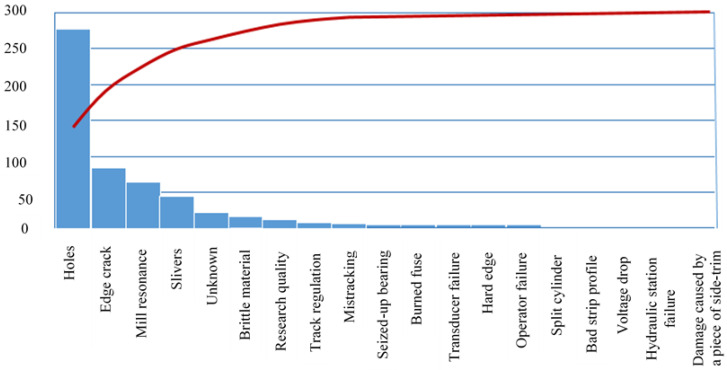
An overview of rupture triggers in steel-strip rolling. In-house database created over a one-year period monitoring strip ruptures in the cold-rolling process on a five-seat tandem.

The first step of the analysis entails examining the rupture trigger. A rupture trigger is a defect that sets off an undesirable incident in the rolling process, resulting in strip destruction. The Pareto chart in Figure 6 shows an overview of the rupture.

The Pareto chart shows initial defects that are rupture triggers. However, a defect listed as a trigger is not the primary cause of the strip destruction in each case. Many times, the final defect is the consequence of process imperfections in the preceding production steps.

Initial defects triggering the strip rupture in the rolling process were extracted from the empirical ruptures database into the Pareto chart. This graphic representation of the analysis clearly shows the main rupture causes. As expected, the most frequent causes were the defects entering the rolling process from the preceding processes. According to evaluation, first among the critical defects were the holes. The holes result from a reduction of the strip, with allocated defects such as sleeves, from the slab casting process. Rolled-in foreign material in the shape of fragments from the hot-rolling guiding equipment was identified as the secondary reason for the emergence of holes. In the hot-rolling process, these fragments are rolled into the strip surface with such intensity that the pickling process is unable to remove them. Fragments of foreign material pose a great risk for the rolling mills.

**Figure 7:**
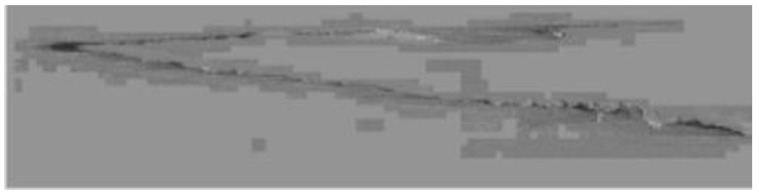
A partially subsurface sliver. Processed in Ametek [30].

**Figure 8:**
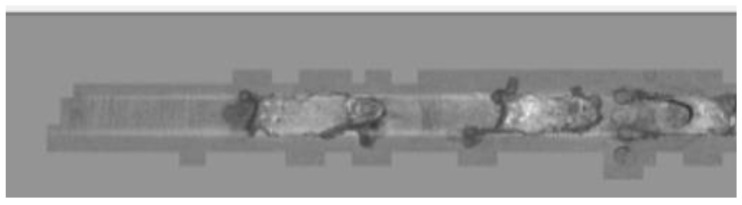
A visible sliver on the strip surface. Processed in Ametek [30].

**Figure 9:**
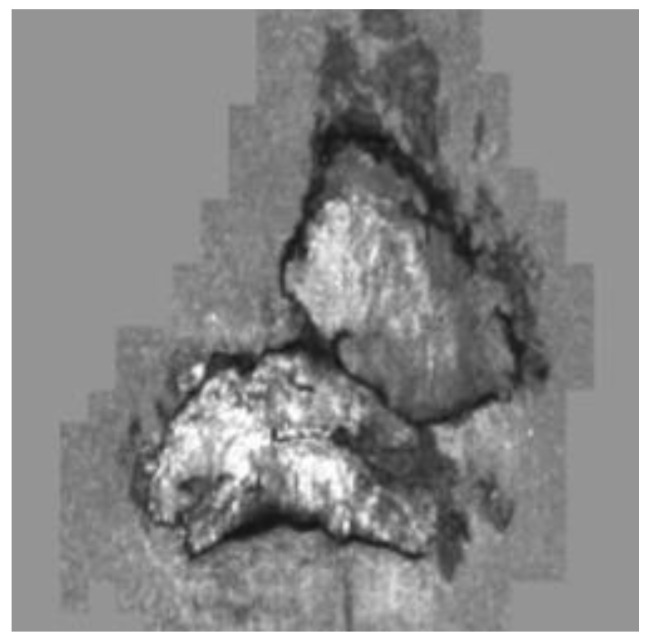
Rolled-in foreign material. Processed in Ametek [30].

Alternatively, slivers are the major cause of the holes. In the technological sequence of production, slivers originate in the process of slab casting, as mentioned in this paper (see Figure 7 and Figure 8). Another critical defect triggering hole formation in the rolling process is rolled-in foreign material, as shown in Figure 9. The theory of hole formation as a result of the said defects has been confirmed by metallographic analyses, which confirmed that in the case of slivers, casting/coating powder elements arranged in a line formation were present in the subsurface steel substrate layers with their origin traced back to the steel-strip production. Where a hole has been confirmed in the rolled material, the metallographic section has shown fragments of a foreign steel material apparently impressed into it. The foreign fragments contained iron oxides and internal oxidation particles, as shown in Figure 9, as well as the metallographic section with rolled-in foreign material.

### 3.2. A Single-Step Defect Detection Method

**Figure 10:**
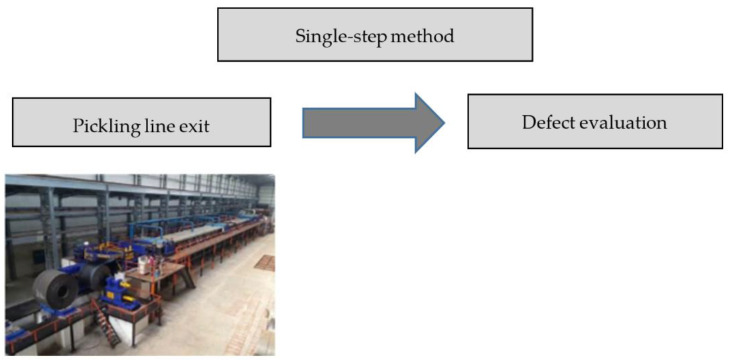
The single-step defect evaluation method chart.

This method enables defect detection prior to rolling only from a single source on a single line, which is usually the pickling line, as shown in Figure 10. Thus, a cross-check of the defect parameters between two technological lines is not possible. This method limits the predictive quality control options in the metallurgical processes, and its use is suitable only in simple metallurgical operations with no flow production.

Advantages of the single-step control are fewer demands on the camera systems, less complex data transfers, no need for comparative computation, fewer requirements on the IT staff, lower costs of camera system installation.

A disadvantage of the single-step control is especially its reduced capability for defect detection, lower quality predictions in the production process, the reduced capability of predictive quality control, and reduced detection capability with respect to deficiencies in the production equipment’s technical condition.

**Figure 11:**
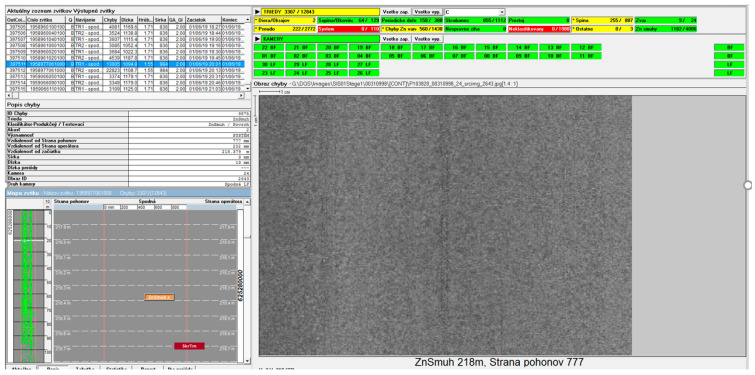
Scratch 5897706—identified after zinc-coated sheet metal.

Figure 11 shows an example of a single-step defect verification method. The defect was captured after the cold-rolling process. The defect occurrence and its effect on the final quality were verified on the next piece of machinery. Figure 11 comes from the zinc-coating line, featuring a Parsytec (ISRA VISION PARSYTEC AG., Aachen, Germany) camera system. The user screen is customized to the Slovak user.

### 3.3. Two-Step Defect Evaluation Method

**Figure 12:**
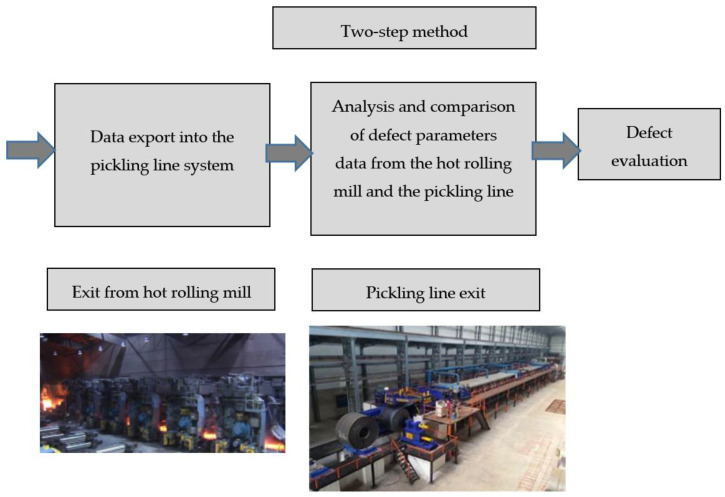
Double-step defect evaluation method chart.

This newly-designed method enables the detection process to start with the initial defect detection in the hot-rolling process and eliminates defects in the production flow between the hot-rolling mill-pickling line and the cold-rolling mill. The basic chart of the two-step defect evaluation is shown in Figure 12.

First and foremost, the exact defect position on the strip surface must be determined to provide for the cross web-coordinate (defect’s position across the strip width) with the strip side defined. Commonly, the strip side is marked to match the direction of the production flow. This is the rolling direction, which is the indexed operator side.

The second parameter determining the defect’s position on the strip is its exact position along the strip’s length, called the down web-coordinate. In order to establish the down web-coordinate, the direction in which the strip moves must be determined.

The third parameter for computing the defect position is to determine which side it occurs on—whether it lies in the upper or the bottom side of the strip. The top-bottom designation changes with the change in the strip’s unwinding/winding.

**Figure 13:**
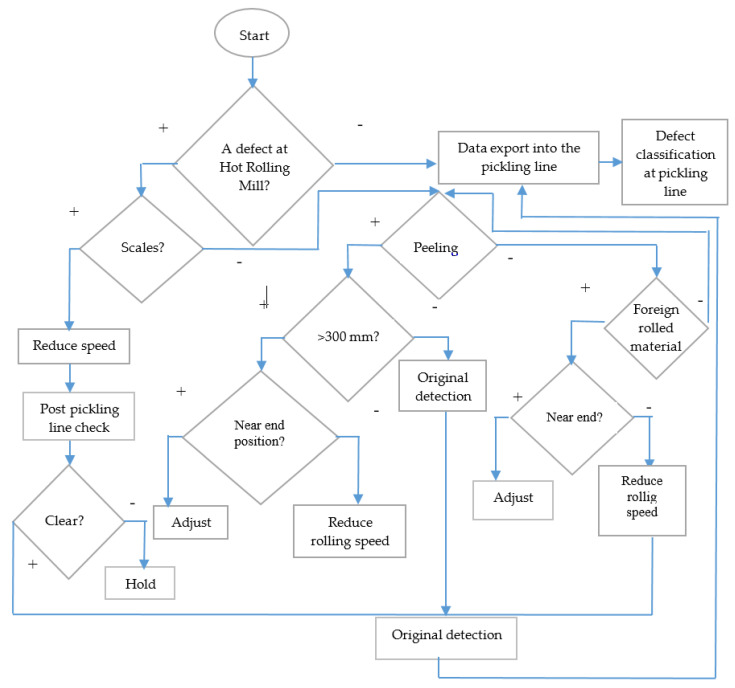
Two-step defect detection flow chart design.

The principle underpinning the two-step defect check on the strip’s surface is to check for the occurrence of a defect in the hot-rolling process and to check it again in the pickling process. The decision-making sequence is shown as the flow chart in Figure 13. As mentioned above, the defect’s exact position must be identified. When the defect is identified in the pickling process, its appearance is compared to the appearance of the defect identified in the hot-rolling process. A mathematical calculation makes it possible to examine the degree of correlation of defects detected in the respective processes. The defect properties are evaluated from the calculation result, as they predict the following characteristics:

- Defect type
- Its effect on the finished product
- Its effect on the subsequent production operation
- Deviations from the standard in the process in which the defect emerged
- Deficiencies in the technical condition of the line in which the defect emerged

The mathematical computation enabling evaluation of the above correlations constitutes an extensive mathematical analysis evaluating defect properties from the defect’s optical characteristics, focusing on the reflexive properties of the defect’s surface, such as the following:

- Number of dark pixels
- Number of light pixels
- Ratio of defect sides
- Light segments width
- Dark segments width
- Defect roundness
- Recurrence
- Defect’s areal span
- Defect’s height
- Defect’s width
- Number of pixels throughout the defect’s dimension
- Comparing the defect’s top-bottom occurrence. If the defect’s position is identical on both sides of the strip, this is a bisurfical defect that poses an increased risk.

#### 3.3.1. Example of the Two-Step Evaluation

**Figure 14:**
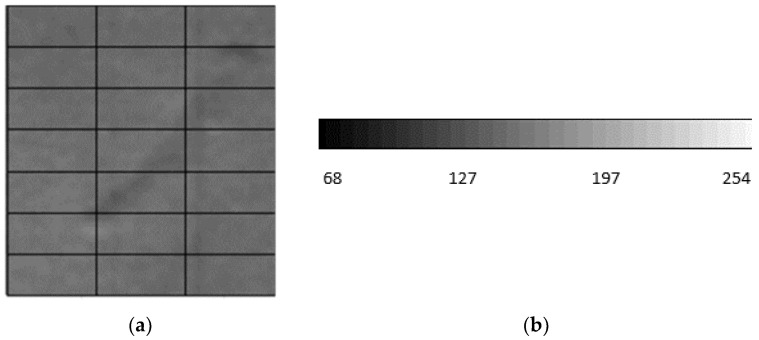
Defect detection of the hot-rolling mill (processed in Ametek): (**a**) defect detected at hot-rolling mill; (**b**) pixels shade scale.

The process of defect-checking on the respective production lines involves calculating the number of pixels making up the defect, its position on the strip, and the defect’s angle with respect to the strip’s axis, i.e., how the defect is positioned on the strip. The pixel shade scale represents the evaluation of the defect’s surface area as the pixel’s color intensity, see Figure 14b. The detected defect surface consists of pixels of various shades ranging from black to white, Figure 14a. The correlation between the two detected objects can be confirmed or rejected based on the result of comparing the number and the shade of pixels from the respective camera systems.

**Figure 15:**
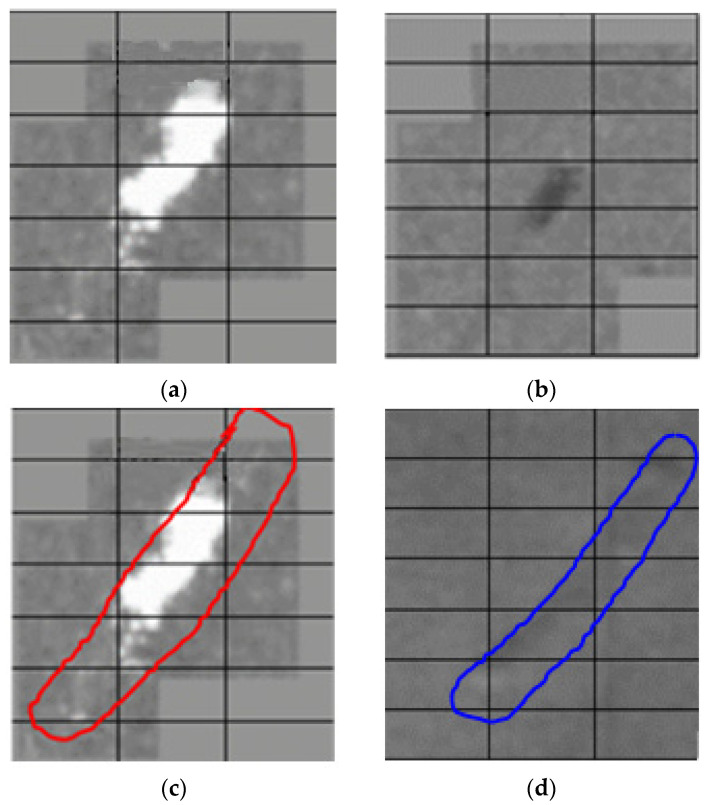
Comparison of the defect in the hot-rolling mill and the pickling line (processed in Ametek): (**a**) Hot-rolling mill check; (**b**) Pickling line check; (**c**) Evaluation of defect’s position with respect to the strip’s axis in the hot-rolling mill; (**d**) Evaluation of defect’s orientation with respect to the strip’s axis in the pickling line.

The process of defect checking at the respective production lines involves calculating the number of pixels filling the defect, its position on the strip and the defect’s angle with respect to the strip’s axis, what is its position on the strip, as shown in the camera footage on Figure 15.

In the application of the two-step method, a defect detected in the hot-rolling mill is checked against the expected position of the defect on the strip in the pickling process. A positive result triggers a classification process and a comparison to show whether the defect matches the one from the hot-rolling mill process.

**Figure 16:**
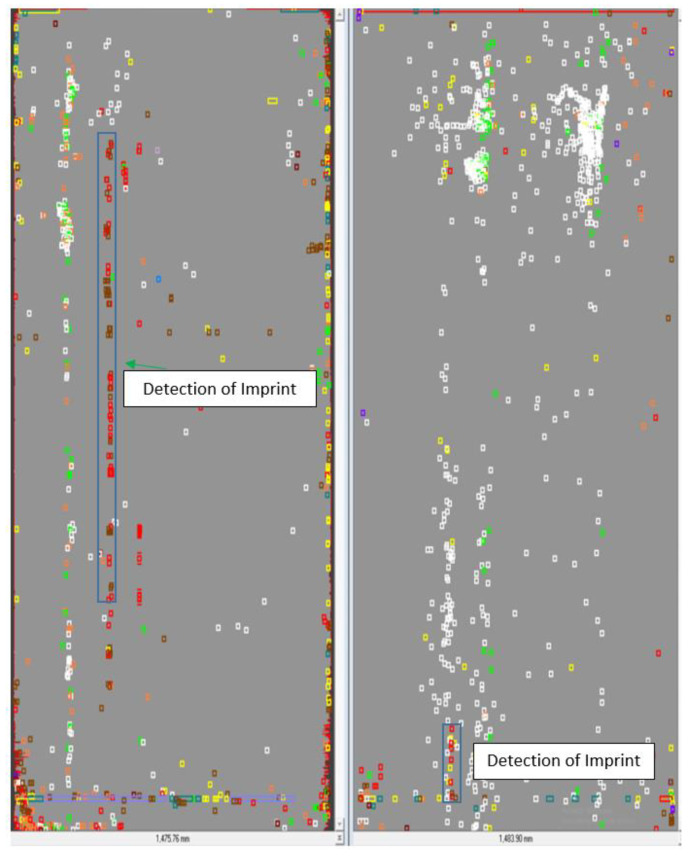
Surface defect distribution Pickling line (processed in Ametek camera), [31]. Left: strip bottom right: strip top.

Figure 16 is from the SmartView Production Quality Advisor application. The individual boxes, i.e., color cubes, mark the defects’ position on the strip. Individual defect types are color-coded. Marked in blue is a line of imprints already detected by the camera system in the preceding step in the hot-rolling mill. This is the principle of the two-step defect verification; that is, defects were detected at the hot-rolling mill and are checked in the pickling line. Some types of defects are eliminated in the pickling process. If the defects are eliminated, the strip is clear and can pass on to the next process.

In the process of position checking, the defect’s position at the hot-rolling mill and its position on the pickling line is calculated—it is the down web-position. At the pickling line, the reverse position value is evaluated due to the counter-unwinding direction of the strip. The mathematical calculation (3) expresses the defect’s position on the strip. In the first step, the position at the hot-rolling mill is determined from a formula where the defect’s position is determined from the observation point of the strip head (head = rolling commencement).

(3) HRMp = Hp

*HRMp—hot-rolling mill position (defect position at the hot-rolling mill)*

*Hp—head position* (defect’s position measured from the strip head all the way down to the defect on the strip at the hot-rolling mill)

The mathematical expression determining the defect’s position in the pickling process is calculated from the defect’s position at the hot-rolling mill. The defect’s position at the pickling line is calculated as follows (4), where the defect’s position at the hot-rolling mill is subtracted from the total strip length, and the result determines the defect’s position on the strip in the pickling process.

(4) PCLp = (Fl − HRMp)

*PCLp*—*pickling line position*

*Fl—full length* (full strip length)

Calculating the defect’s cross-web position on the strip is done in the direction of the strip’s unwinding, and when it is evaluated at the pickling line, its value is opposite the value it has at the hot-rolling mill due to the strip’s reverse direction in the pickling process.

Should there be an 85% match between the defects evaluated in the calculation, the defects detected at two different production lines may be considered identical and they may be evaluated in terms of the defect type and its impact on the subsequent production process steps affecting the quality and customer requirements. Should the type, the severity and the scope of defect be inadmissible, the defect is removed from the strip. Should the defect fit the admissible requirement limits, the product is released to continue down the production flow and eventually to the customer.

#### 3.3.2. Recommendations of the Two-Step Evaluation

Marking the side of the strip where the defect is located is done subject to the spatial layout of the production line and the process owner’s requirements. Usually, the sides are marked as left or right, while it is important that such marking respects the direction of the strip unwinding. Alternatively, the drives’ side is used to mark the strip sides (the side of the defect on which the line’s drives are located) or the operator’s side (that side where the line controls are located).

Important for determining the defect position evaluation side is the placement of cameras and determining the respective strip side in the camera system. Strip surface defect analysis and defect comparison and classification in the preceding processes is the key parameter in the correct defect analysis.

In designing and implementing the plants of the future, it is necessary to account for working nodes and equipment layout in a progressive manner and to design the workspace and the material flow efficiently, together with the analysis of the need for support activities crucial for efficient and economical operation of the means of production.

An elementary step in plant designing is planning for an ideal state of the technological line to ensure maximum production process efficiency. An ideal state is often limited by spatial, technological, financial, and legislative constraints.

In planning the technological flow, it is necessary to define the processes that will be monitored and the exact locations of data harvesting, i.e., individual readers directly in the process, which means monitoring points for yielding direct process data. Further, it is necessary to establish the individual production process parameters to be calculated and the data to be stored in databases. The databases that will provide the data for the direct process must be available in real-time and the data for the process analysis or for documenting the history or reverse process verification may be stored in external data storage. External data storage offers the company the opportunity of storing a large volume of data under favorable conditions and the manufacturer itself does not, therefore, have to expend resources on data storage and management, which contributes to significant cost reductions for data storage and management.

Further development of the predictive quality management systems should aim at increasing the accuracy of the strip surface defect detection, thus increasing the effectiveness of predicting negative effects. The last but not least goal of the further SMART systems development is its focus on predicting the technical condition of the production systems and predicting the necessary maintenance interventions on the technological equipment, thus increasing its lifespan and reducing the costs related to failures in the production process.

## 4. Discussion

The paper presents an innovative predictive system of quality management involved in the processes of metallurgic production. This system is a highly effective tool for ensuring a sophisticated production process of metallurgic products, in particular sheet steel rolls. The principle idea behind this innovative system is predictive quality management in the production process, which means to be a step ahead of compromised quality and, at the same time, to eliminate the losses caused by the process imperfections. The predictive system of quality management described above is implemented into the existing production process, where modern camera systems were installed, as they are at the core of the said system. Application into an existing production line is possible. However, an ideal solution is to install sophisticated support systems into newly-built production lines, where the necessary installations are custom-designed according to the newly-built production system requirements.

The predictive system presented here draws on the defects’ optical properties detected on the strip surface and uses mathematical calculation for their classification. Currently, the principle of defect detection and classification is applied first and foremost to those that are classified in the hot-rolling process. Defect classification on the hot-rolling mill is the basis for a two-step system of defect detection and classification. The defect is classified according to its optical properties and it is checked at the exit from the pickling line and compared to the defect detected at the hot-rolling mill. If its expected properties match those predicted for the expected effect, it is possible to establish the defect type and the extent of its effect on the product and on the subsequent production processes. Based on the defect’s evaluation, a decision is taken on its further processing or rework with the aim of maximum product utilization and minimum negative effects of the defect on the production equipment and elimination of adverse events or failures in the production process.

**Figure 17:**
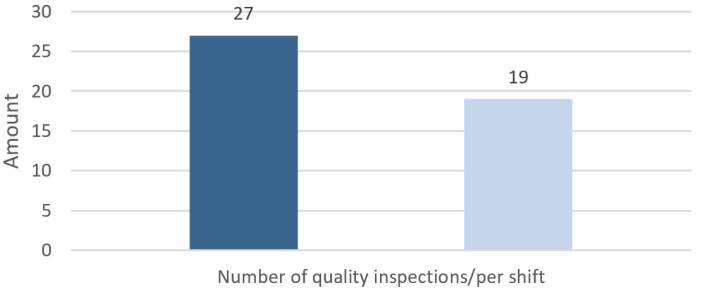
Comparison of the number of strip surface quality inspections over a 12-h shift before and after the introduction of the two-step quality control.

Below is a practical example of the possible reduction in the number of quality inspections over a 12-h shift at the cold-rolling mill (Figure 17).

**Figure 18:**
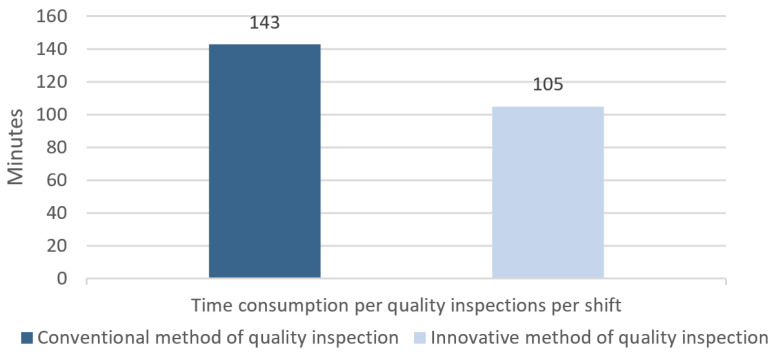
Comparison of an average strip surface quality inspection time over a 12-h shift before and after the introduction of a two-step quality control.

As Figure 18 shows, the new, innovative approach not only results in fewer quality inspections but also in the reduced time needed for such an inspection. Thus, the productivity of work is significantly improved.

## Data Availability

Not applicable.

## Appendix A

PDI data telegram that enters the rolling track with the incoming coil, Figure A1.

1. Coil ID—coil designation, 19* manufacture year, *40211* meltage number, *03* the order in which the coil appears in the particular meltage (10–12 slabs, i.e., coils are obtained from one meltage), *0100 mother piece after it has been rolled in the hot-rolling mill (if it is joined with other piece or the mother piece splits, the last digits are 1000, 1100, 1200, etc.)
2. PROCESS 4—further production direction (cold-rolling mill)
3. AIM GAUE “0.905”—output strip thickness in millimeters tolerance ±0.070 mm
4. OPERATION = B—cold-rolling operation
5. INCOMING_COIL_LENGHT—length of the incoming coil
6. HOT BAND AVERAGE GAUGE 3041—hot strip thickness, max 3.17 mm, min 2.83 mm
7. HOT BAND COILING TEMP 612.683—coiling temperature at the hot-rolling mill
8. HOT BAND FINISHING TEMP—Nom. 845 degrees, Max 870, Min 820, Finishing temperature at the hot-rolling mill
9. GAUGE_TOLERANCE_PLUS = 07.735—Width tolerance
10. GAUGE_TOLERANCE_MINUSS = 07.735—Width tolerance
11. GRADE = 0589—technological code -steel quality class-kind
12. COIL_ENTRY_WEIGHT = 17,620—incoming coil weight in kg
13. COIL_WIDTH = 1275.00000—coil width in mm
14. NEXT_OPERATION = 0222—next operation with a side trim
15. HOT CROWN = 0.0918 mm—hot profile convexity, max 0.0900 mm, min 0.040 mm
16. SHIM SIZE = 0.0178 mm—hot strip wedging max ± 0.030 mm
17. WELD_DIST 1 = 0—weld distance from the strip’s beginning (if the value is 0 there is no weld -the pickling line weld is meant in such case)
18. WELD_DIST 2 = 0—weld distance from the strip’s beginning (if the value is 0 there is no weld—the pickling line weld is meant in such case)
19. WELD_DIST 3 = 0—weld distance from the strip’s beginning (if the value is 0 there is no weld—the pickling line weld is meant in such case)
20. FLATNES_TARGET_INDEX = 4—rolling curve value
21. FLATNES_TARGET_AMPLITUDE = 30—rolling amplitude value
22. COSTUMER = N—customer undesignated
23. WELD_RELIABILITY = WELD QUALITY—(if the value is 0 the coil is weld-free)
24. FINAL_OPERATION = 6710—final machinery, in this case, 6710 is the galvanizing line
25. Chemical composition of steel—(publishing prohibited)
